# Media exposure to COVID-19 information, risk perception, social and geographical proximity, and self-rated anxiety in China

**DOI:** 10.1186/s12889-020-09761-8

**Published:** 2020-11-04

**Authors:** Miao Liu, Hongzhong Zhang, Hui Huang

**Affiliations:** College of Journalism and Communication, Beijing Normal University, Xinwai Dajie 5 Hao, Haidian District, Beijing, China

**Keywords:** Self-rated anxiety, Media exposure, Social proximity, Geographical proximity, Risk perception

## Abstract

**Background:**

The coronavirus disease 2019 (COVID-19) is an emerging infectious disease that spreads around the world. The lack of effective antiviral drugs and vaccines, along with the relatively high mortality rate and high contagiousness, has raised strong public concerns over COVID-19, especially for people living in the most severely affected areas. This study aimed to clarify the influencing factors for the anxiety level among the Chinese people during the COVID-19 pandemic, with a particular focus on the media exposure to different COVID-19 information.

**Methods:**

A total of 4991 respondents were randomly recruited from a national online panel from February 12th, 2020 to February 14th, 2020, a period when the number of COVID-19 cases surpassed 10,000 in a single day, with the total cases in China reaching up to 90,000. The relationships between media exposure of COVID-19 information, social and geographical proximity to COVID-19, risk perceptions were assessed using hierarchical ordinary least squares regression analysis.

**Results:**

The media exposure to COVID-19 information was differently associated with anxiety. Meanwhile, the anxiety level was found to be high in respondents who personally knew someone infected with COVID-19 or those who living in an area with reported cases. Respondents who perceived more risks also reported a higher level of anxiety.

**Conclusions:**

This study highlights the role of media exposure in affecting individuals’ anxiety level during the COVID-19 pandemic. Besides, it is recommended that government and health professionals are recommended to adopt effective risk communication strategies to protect citizens’ mental health during the pandemic.

## Background

The coronavirus disease 2019 (COVID-19) is an emerging infectious disease that quickly spreads around the world. The outbreak began in Wuhan in December 2019, since then, more than 90,000 cases have been confirmed in China so far. The newly-found disease has killed over 4700 people in China and 800 thousand people globally till August 2020. According to the World Health Organization (WHO), COVID-19 is related to the SARS-associated coronavirus (SARS-CoV) that caused an outbreak of severe acute respiratory syndrome (SARS) in 2002–2003 [1]. The typical symptoms of COVID-19 include cough, fever, and shortness of breath. People who are infected with COVID-19 can transmit the virus to others even when they do not display any symptoms [2]. On January 30th, 2020, the WHO declared the outbreak in a public health emergency of international concern [3]. Due to the high infectiousness and “non symptom” characteristics, COVID-19 has raised great anxiety and panic among the Chinese population.

The media-disseminated information has played a crucial role in affecting the risk perception and anxiety of the public during a pandemic [4]. At the beginning of the COVID-19 outbreak, the public were not quite concerned about the severity of this novel coronavirus, because they were misguided by the information that “there has been no evidence of human-to-human transmission” released by the Wuhan Municipal Health Commission [5], and such information was then widely circulated on both mass media and at social networking site. The public awareness about the seriousness of COVID-19 changed when Zhongnanshan, the head of the National Health Commission’s team, appeared in the state-owned media and confirmed the human-to-human transmission of this novel coronavirus on January 20th, 2020. Thereafter, COVID-19 quickly became a national health emergency, and the viral infection spread to every province in less than two weeks. Wuhan, the epicenter of the novel coronavirus outbreak with the official population of over 11 million, was shut down on January 23rd, 2020, marking the biggest government intervention to stop COVID-19 spreading. Soon after that, nearly all the Chinese people were quarantined at home. During the COVID-19 outbreak, the Chinese people received tons of information about COVID-19 through various outlets.

Up until now, the COVID-19 vaccine is still underway. The lack of effective antiviral drugs and vaccines, along with the relatively high mortality rate and infectiousness, has raised strong public concerns, especially for those people who are living in the most severely affected areas. During the quarantine period, people are filled with fear and anxiety over the COVID-19 pandemic, as well as panic over scarce resources [6, 7]. The exposure to COVID-19 information in media inevitably affects the public’s responses to the pandemic, since media serves as the main information source during the quarantine period. Dissemination of accurate and credible information about COVID-19, particularly the prevention measures, could effectively slow down virus spreading and mitigated disease-associated apprehension across the population. There are different findings from previous research concerning the effect of media exposure on anxiety during the outbreak of a pandemic. For example, one study found that the media created little anxiety among the Canadian college students a few months after SARS outbreak [8]. However, another study revealed that more television exposure is related to higher level of anxiety during H5N1 across the European Union [9]. Therefore, it is difficult to generalize the effect of media exposure on the psychological responses, such as anxiety.

A handful of research has been conducted to examine the public’s anxiety level during the pandemic, such as ebola [10], H7N9 [11] SARS [8], and H5N1 [12]. These studies have generally come to the conclusion that the outbreak of a pandemic is associated with a higher level of anxiety among the public. In this regard, it is important to understand the psychological factors that predict anxiety during the outbreak of a pandemic, because health anxiety may lead to clinically significant distress (e.g., anxiety and fear), worry, and excessive preventative behaviors such as excessive hand washing, repeatedly seeking reassurance from medical professional in some people [13].

To date, there is little research focusing on the effect of media exposure to different contents on the anxiety level during a global pandemic. Therefore, it is valuable to elucidate the factors contributing to anxiety during a pandemic, so as to understand the public responses to a health emergency of international concern more generally and to identify the possible maladaptive responses of individuals [3]. The present work aimed to identify the factors associated with anxiety during the outbreak of COVID-19, with the particular focus on the effect of media exposure.

## Methods

According to estimates, landline telephone only covers 182 million households in China [14], and there is a large amount of population that cannot be accessed using traditional landline-lined based telephone surveys. By contrast, there are 854 million Chinese people getting access to Internet in 2019 [15]. Therefore, an online sample collection method was adopted in the present study. Upon IRB approval, 6892 respondents were randomly recruited from a national online panel, named Acadeta (databnu.com), which consists of 1,075,809 Chinese adults, from February 12th, 2020 to February 14th, 2020. During this period, the number of COVID-19 surpassed 10,000 in a single day, with the total cases in China reaching up to 63,940. After the data cleaning procedure, 4991 respondents remained in the sample. The response rate of the present study was 18.67%. All the respondents completed the online informed consent form before filling out the survey.

### Risk perception

Respondents were asked to assess their perceived risks of “being affected by the novel coronavirus” and “being affected by the novel coronavirus compared with others,” which were measured on a 5-point Likert-type scale ranging from 1 (very unlikely) to 5 (very likely).

**Anxiety level** was measured by the Chinese version of Zung Self-Rating Anxiety Scale (SAS-20) [16] consisting of 20 questions. Respondents were asked to estimate their anxiety levels within a period of one or 2 weeks prior to taking the test. The sample items included: “I feel afraid for no reason at all”; “I can feel my heart beating fast”; and “I have nightmares.” Responses was rated on a 4-point Likert-type scale anchored at 1 (never or very rare), 2 (sometimes), 3 (often), and 4 (very often or always). The total SAS score might vary from 20 (no anxiety at all) to 80 (severe anxiety). Zung recommended converting the raw score to the Index Score (range: 25–100) by multiplying the raw score by 1.25. The index score of 50 and above was used as the cut-off point. The internal reliability (Cronbach alpha) was .84 (*M* = 42.05, *SD* = 10.07).

**Social proximity** was measured by the dichotomous question “Is there someone you know who is affected by the novel coronavirus?” with 0 = No and 1 = Yes. **Geographical proximity** was measured by the direct dichotomous (yes/no) question “Are there reported cases of infection in your neighborhood or in your town?”

### Media exposure

To assess the amount of media exposure to different COVID-19 related contents, we synthesized 10 categories of COVID-19 information that frequently appear in mass media and social media. Respondents were asked to indicate how much attention they paid to the each of the following information in media, including “how to prevent COVID-19 infection,” “the number of infected cases,” “news coverages of patients,” “news coverage of doctors and nurses,” “news coverages of government officials,” “news coverages of scientists,” “donation information,” “life of ordinary people during the COVID-19 outbreak,” “information about returning to work/school,” and “analysis of the pandemic” (1 = Do not pay any attention at all; 5 = Pay a lot of attention).

### Data analyses

The data were analyzed using SPSS 25.0 software. Pearson correlation analysis was conducted to examine the relationships between predictors and the outcome. According to central limit theorem, the sampling distribution is assumed to approach normal distribution considering that the present study has a large sample size (*n* = 4991). Before conducting the correlation analysis, scatterplot was employed to check the linearity and the variables included in the analysis exist linear relationships. The level of significance was set at *p* < 0.05. Hierarchical ordinary least squares (OLS) regression analysis was utilized to assess the associations between anxiety, media exposure, social proximity, geographical proximity, and risk perceptions, adjusting for age, sex, education, and income.

## Results

Table 1 depicts the demographics of the respondents and the descriptive data for key variables. Of the respondents, 18% had high school education or lower, 31.9% had associate degree, 47% had college degree, and 5% had graduate degree. Meanwhile, 49.6% of the respondents were male while 50.4% were female. A majority of the respondents had the monthly income below 5000 RMB. Among the 4991 respondents, only 5.87% reported that they knew someone who were infected with COVID-19 personally. 32.2% indicated that there were reported cases in the area where they live. 5.9% of the respondents perceived their likelihoods of “acquiring COVID-19” as high or very high, while over half of the respondents rated their risks of acquiring COVID-19 as low. The anxiety levels in most respondents fell in normal range (M = 42.05; range 25–100), 14% reported mild to moderate anxiety level, 5.1% of the respondents experienced moderate to severe levels of anxiety, and only 1.5% reported severe anxiety. As shown in Table 1, 62.7% of the respondents received COVID-19 information through WeChat, 60.8% through television, 45% via tiktok, 42.4% by interpersonal communication, 38.9% by Weibo (a twitter-like social media), 15.5% via newspaper, and 8.9% through radio. A majority of the respondents (65.2%) consider there are “some rumors” in their information environment. Table 2 shows the mean and standard deviations of the amount of media exposure to different COVID-19 information. Table 3 presents the correlations between demographic variables, social and geographical proximity, perceived risks, and anxiety. The results revealed that self-rated anxiety is significantly associated with most independent variables.

**Table 1:** Respondents’ characteristics, information outlets, information perception, social and geographical proximity, anxiety level (*N* = 4991)

Demographics	N	%
Gender		
Female	2514	50.4%
Age		
18–30 years	3203	64.2%
31–40 years	1246	25.0%
41–50 years	399	8.0%
51–60 years	126	2.5%
≧61 years	17	0.3%
Education		
Primary or secondary school	800	16.0%
Associate degree	1585	31.8%
College	2356	47.2%
Graduate degree	250	5.0%
Monthly Income		
≦1000 rmb	929	18.6%
1001–2000 rmb	361	7.2%
2001–5000 rmb	1707	34.2%
5001–8000 rmb	1498	30.0%
8001–15,000 rmb	411	8.2%
≧15,001 rmb	85	1.7%
Anxiety Level		
Normal (< 50)	3963	79.4%
Mild to moderate anxiety levels (50–59)	699	14%
Moderate to severe levels (60 to 69)	256	5.1%
Severe anxiety levels (> 70)	73	1.5%
Social Proximity		
Yes	293	5.9%
Geographical Proximity		
Yes	1608	32.2%
Information Outlets		
Television	3033	60.8%
Radio	442	8.9%
Newspaper	772	15.5%
Interpersonal	2114	42.4%
Wechat	3130	62.7%
Weibo	1941	38.9%
tiktok	2246	45%
Online news website/news app	3926	78.7%
Search engine	1390	27.9%
Information Perception		
Only a little rumors	560	11.2%
Some rumors	3254	65.2%
A lot of rumors	1177	23.6%

**Table 2:** Exposure to different COVID19 related information

Information exposure	M(SD)
How to prevent COVID-19 infection	4.00(.88)
The number of infected cases	4.06(.92)
News coverage of patients	3.71(.99)
News coverage of doctors and nurses	3.71(.99)
News coverage of government officials	3.46 (1.07)
News coverage of scientists	3.68 (1.05)
Donation information	3.33 (1.02)
Life of ordinary people during the COVID-19 outbreak	3.61(.99)
Information about returning to work/school	3.84 (1.08)
Analysis of the pandemic	3.91(.99)

**Table 3:** Pearson correlations between perceived risks, social and physical proximity to COVID-19, self-rated anxiety

	1	2	3	4	5	6	7	8
1.Gender								
2.Age	−.002							
3.Education	−.03*	.05*						
4.Income	.12**	.44**	.32**					
5.Perceived risk	−.004	−.03	.04*	.01				
6.Perceived risk compared to others	−.03	−.01	.05**	−.01	.65**			
7. Social proximity	.003	.07**	.05**	.06**	.12**	.14**		
8. Geographical proximity	−.02	−.05**	−.07**	−.07**	.18**	.19**	.20**	
9. Self-rated anxiety	.03	−.12**	.03	.004	.22**	.20**	.14**	.09*

*p < .05 ** *p* < .01

Table 4 presents the results obtained from regression analysis that predicts the self-rated anxiety. Demographic variables including sex, age, gender, and education were entered in the first block, followed by social proximity and geographical proximity, while perceived risk and perceived risk compared with others were entered in the third block, and exposure to different COVID-19 information in the fourth block. Among the demographic variables, the results revealed that education was significantly associated with the perceived anxiety (*β* = .04, *p* <. 01), with respondents who have higher education reported more anxiety. Meanwhile, age was found to be negatively associated with perceived anxiety (*β* = − .14, *p* <. 001), with the younger respondents experienced a higher level of anxiety than the older respondents. Gender and income were not significantly associated with anxiety. Taken together, the demographic variables accounted for 2.1% of the total variance.

**Table 4:** Ordinary least squares regression analysis predicting self-rated anxiety

	Self-rated Anxiety			Model 4
	Model 1	Model 2	Model 3	
Variable	beta	beta	beta	beta
Demographic				
Gender	.02	.03	.03	.01
Age	−.14***	−.14***	−.14***	−.11
Education	.05**	.05*	.04*	.04**
Income	.03	.03	.03	.04**
Proximity				
Social Proximity		.14***	.12***	.11***
Geographical Proximity		.05**	.02	.02
Perceived risk				
Perceived risk			.13***	.12***
Perceived risk compared to others			.07***	.08***
Information exposure				
How to prevent COVID-19 infection				−.13***
The number of infected cases				−.09***
News coverage of patients				.01
News coverage of doctors and nurses				.02
News coverage of government officials				.003
News coverage of scientists				−.03
Donation information				.12***
Life of ordinary people during the COVID-19 outbreak				.08***
Returning to work/school				−.08***
Analysis of the pandemic				−.08***
Adjust *R* squared	.02	.05	.08	.14
ANOVA	*F*(4,4990) = 24.88	*F*(4,4990) = 35.71	*F*(4,4990) = 34.49	*F*(4,4990) = 41.24

*p < .05 ** *p* < .01 ***p < .001

After adjusting for demographic variables, social proximity and geographical proximity were positively associated with the self-rated anxiety (*β* = .14, *p* < .001 and *β* = .05, *p* < .001, respectively). Respondents who personally knew someone infected with COVID-19 or those who lived in a neighborhood with reported COVID-19 cases also experienced a higher level of anxiety. Social proximity and geographical proximity accounted for an additional 2.3% of the total variance.

Further, the perceived risk of COVID-19 (*β* = .13, *p* < .001) and perceived risk of COVID-19 compared with others (*β* = .08, *p* < .001) were both positively associated with the perceived anxiety. The perceived risks accounted for an additional 3.2% of the total variance.

As for the relationship between media exposure of COVID-19 information and the self-rated anxiety, our results revealed that information about “how to prevent COVID-19 infection,” “the number of infected cases,” “information about returning to work/school,” and “analysis of the pandemic” were negatively associated with self-rated anxiety. “Donation information” and “life of ordinary people during the outbreak” were positively associated with self-rated anxiety. Moreover, “news coverage of patients,” “News coverage of doctors and nurses,” and “news coverage of scientists” were not significantly associated with self-rated anxiety. Together, media exposure accounted for 5.1% of the total variance in self-rated anxiety.

## Discussion

This study preliminarily investigated on the public’s anxiety level during the outbreak of COVID-19 in China, which is one of the biggest infectious disease outbreaks in Chinese history. One notable strength of the present study was that the data were collected at the peak of COVID-19 outbreak in China. Thus, the public responses to COVID-19 were more accurately recorded than retrospective reporting. Generally, it was found that the anxiety levels of most respondents were normal during the outbreak of COVID-19. Even though the data were collected at the peak of COVID-19 outbreak, a majority of respondents reported low to medium level of perceived risks. One possible explanation for these findings is that most people were mandated to be quarantined at home, which might lower the risk perceptions and anxiety over COVID-19 infection. Most respondents considered that they themselves knew the COVID-19 fairly well.

Media played a particular important role in affecting the public responses to COVID-19 as most Chinese people were quarantined at home to prevent the possible virus spreading during the outbreak. As a result, the people spent a large amount of time on media. Previous research has generally found that the increased exposure to pandemic-related information leads to a higher level of anxiety [13]. These studies commonly measured media exposure by assessing the amount of information that people received from the media during the pandemic, while little is known about what the specific type of information that may increase or decrease anxiety. The present study focused on the effect of media exposure to information about COVID-19 on the anxiety level. National media, social media, the China CDC, and government authorities, disseminate information in a timely manner to make people stay informed during the pandemic. It is thus virtually impossible that people are not exposed themselves to any COVID-19 information at all. The main finding of the present study is that media exposure to different COVID-19 information influenced people’s self-rated anxiety in distinct ways. The results showed that some COVID-19 information increased public anxiety, while others decreased it. For example, it was found that donation information and the life of ordinary people were positively with self-rated anxiety. One possible explanation was that donation information was a signal that the hospitals were in shortage of protective equipment, which might therefore increase the public anxiety level. During the pandemic, most news coverage about the life of ordinary people were negatively-valanced, which focused on the stories of people who were infected with or died of the novel coronavirus. Therefore, exposure to information about the life of ordinary people increased people’s anxiety level. Information about returning to work/school was negatively associated with the anxiety level, because it suggested that the pandemic was under control and quarantine was soon be over. Interestingly, information about the number of reported COVID-19 cases and the analysis of the COVID-19 pandemic were negatively associated with anxiety. It was speculated that information regarding the numbers of reported cases and deaths actually lowered the uncertainty level of the public, because uncertainty often arises with lack of information and “uncertainty is experienced subjectively as anxiety” [17]. Compared with those who do not know the real situation of the pandemic, people are likely to feel less anxious when they get to know more information about the seriousness of the pandemic through media. Not surprisingly, information about how to prevent COVID-19 was negatively associated with the level of anxiety in this study, because knowing more about the prevention of COVID-19 could lower people’s anxiety over the novel coronavirus infection.

It was hard to draw a strong causal conclusion about the relationship between media exposure and anxiety based on results in this study. Yet it seemed to be reasonable to assume that different media contents had different impacts on people’s anxiety level, since a majority of participants (72.1%) didn’t actively search for COVID-19 information using web search engine. Despite that social media afford users some level of flexibility in choosing the content, in general, most people are the passive social media consumers who only acquire information on their timeline. Therefore, it is not easy for people to get themselves exposed to the content-specific COVID-19 information because most participants receive information in a more passive manner.

Overall, the more educated and younger respondents experienced a relatively higher level of anxiety. Such results were generally consistent with the previous research suggesting that young people were more likely to be anxious than the older adults [18]. Previous studies have found that disease knowledge is a significant predictor of anxiety level [19, 20]. This may be ascribed to the fact that the young people and people who are more educated receive more information about COVID-19 through different media outlets than older adults and people who are less educated, so they are more likely know the severity of contracting COVID-19. Therefore, younger and more educated people who have more knowledge about COVID-19 are more likely to feel anxious. Gender and income did not have significant associations with anxiety level.

Another important finding generated from the present study was that, both social proximity and geographical proximity to COVID-19 were positively associated with anxiety. Due to the high infectiousness of COVID-19, people who personally knew someone infected with COVID-19 were more susceptible to anxious thoughts than others. Living in an area that had reported cases was also positively associated with anxiety. The results were consistent with the previous study showing that a shorter distance to the risk resulted in higher risk perception [21]. Moreover, individuals who perceived more risks reported a higher level of anxiety than those who perceived less risks. It is possible that people who have higher level of risk perception overestimate their risk of COVID-19 infection and therefore feel more anxious. Nevertheless, to better understand the causal relationship between anxiety and risk perception, longitudinal studies are recommended.

Our study has several limitations that are worth noting. Firstly, our results might suffer from generalizability problem. Besides, the online panel inherently came with sample selection bias. The respondents were generally younger and more educated in our sample, and may therefore not be representative of rural populations in China. At present, a majority of Chinese citizens can get access to the Internet, but there are still people who are unable to access to Internet, especially for the older adults or those living in the economically disadvantaged areas. Moreover, this study was unable to explore how the level of anxiety fluctuated from the beginning of the pandemic to the time of data collection, therefore we cannot make strong causal inference due to the cross-sectional nature of this study.

This study raises important implications. Government and public health practitioners are recommended to take prevention-focused approach to promote citizens’ mental health during the pandemic. If the public’s psychological well-being cannot be ensured, heightened emotions such as anxiety could lead to detrimental social effects. For example, one study found that increased anxiety may lead to shortage of medical supplies such as face masks [11].

## Conclusions

In conclusion, our findings suggest that anxiety level is differently associated with media exposure to COVID-19. The innovativeness of this study is its specific focus on the effect of different media contents on people’s psychological response in the context of China. The anxiety level was also explained by social proximity and geographical proximity to COVID-19, as well as perceived risk. Since the narratives of media play a major role on the public responses to COVID-19, government and media professionals are recommended to deliver the balanced information about the pandemic instead of overemphasizing the negative information, which may fuel panic and uncertainty among the public. Furthermore, dissemination of positive-toned information in media, such as infection prevention of the novel coronavirus, can be the effective anxiety management tools. Overall, the present study adds great value in understanding the public responses to different media contents that are under strict censorship by the Chinese government in a media-saturated age.

## Data Availability

The dataset used and/or analyzed during the current study are available from the corresponding author on reasonable request.

## Abbreviations

COVID-19: Coronavirus disease 2019
WHO: World Health Organization
SARS: Severe acute respiratory syndrome
China CDC: China Centers for Disease Control and Prevention
Ebola: Ebola Virus Diseases
H7N9: Asian lineage avian influenza A
H5N1: Highly Pathogenic Asian Avian Influenza A
